# The neurosurgical outcome of pediatric Cushing’s disease in a single center from China: a 20-year experience

**DOI:** 10.3389/fendo.2025.1663624

**Published:** 2025-09-03

**Authors:** Baofeng Wang, Hongchi Zhang, Tingwei Su, Jie Ren, Qingfang Sun, Yuhao Sun, Liuguan Bian

**Affiliations:** ^1^ Department of Neurosurgery, Ruijin Hospital, Shanghai Jiao Tong University School of Medicine, Shanghai, China; ^2^ Department of Endocrinology, Ruijin Hospital, Shanghai Jiao Tong University School of Medicine, Shanghai, China; ^3^ Department of Neurosurgery, Ruijin Hospital Luwan Branch, Shanghai Jiao Tong University School of Medicine, Shanghai, China

**Keywords:** Cushing’s disease, pediatric, transsphenoidal surgery, surgical outcome, surgical strategy

## Abstract

**Objective:**

Pediatric Cushing’s disease (CD) is exceptionally rare and poses significant diagnostic and therapeutic challenges. This study aimed to review the diagnostic features and to evaluate the long-term surgical outcomes of transsphenoidal surgery (TSS) in Pediatric CD patients at a single tertiary center in China over two decades.

**Methods:**

A retrospective analysis included 22 pediatric CD patients (10 male, 12 female; mean age 15.8 ± 2.5 years) who underwent TSS between 2002 and 2022. Diagnosis was established through a multidisciplinary protocol involving standardized biochemical testing (LDDST, HDDST), bilateral inferior petrosal sinus sampling (BIPSS) with desmopressin stimulation (n=19), and high-resolution pituitary MRI. Microscopic TSS (MTSS) was performed before 2016 (n=11) and endoscopic TSS (ETSS) thereafter (n=11). Surgical strategy was guided by MRI and BIPSS findings. Immediate remission was defined as a postoperative serum cortisol nadir <5 μg/dL or normal 24-h urinary free cortisol (UFC). Recurrence was defined as the reappearance of hypercortisolism after remission. Mean follow-up was 29.4 months (range 2-129).

**Results:**

MRI identified the adenoma in 18/22 patients (81.8%; 16 microadenomas, 2 macroadenomas). BIPSS indicated lateralization in 14/19 patients (73.7%), with concordance between BIPSS and MRI lateralization in 57.9% (11/19) of cases. Immediate postoperative remission was achieved in 20 patients (90.9%). The two non-remitters (one macroadenoma, one MRI- and pathology-negative) received additional therapies. Among the 20 patients with initial remission, 2 (10.0%) developed recurrence (one microadenoma, one MRI-negative) during follow-up. The sustained long-term remission rate was 81.8% (18/22).

**Conclusion:**

Transsphenoidal surgery represents a highly effective first-line treatment for pediatric CD, achieving high rates of immediate (90.9%) and long-term remission (81.8%) in a specialized center. A meticulous diagnostic approach incorporating BIPSS is crucial, particularly for MRI-negative cases. While recurrence occurred in a minority of patients, primarily those with microadenomas, durable disease control is attainable for the majority with appropriate surgical management. The transition to endoscopic techniques was feasible and effective.

## Introduction

Cushing’s disease (CD), caused by excessive ACTH secretion from a pituitary corticotroph adenoma, is a rare disorder with an estimated prevalence of approximately 10 cases per 100,000. Its incidence is even lower in children, representing about 5% of adult cases (1). CD accounts for 75-80% of Cushing’s syndrome in pediatric patients (2, 3). Clinical manifestations include weight gain, facial rounding (“moon facies”), hypertension, fatigue, and pubertal arrest. If untreated, pediatric CD can severely impair quality of life and lead to significant morbidity and mortality.

Diagnosis of pediatric CD is frequently delayed due to atypical symptoms and remains significantly challenging for pediatricians and pediatric endocrinologists (4). It relies on standardized biochemical evaluation and neuroimaging. Transsphenoidal pituitary surgery (TSS), encompassing both microscopic and endoscopic approaches, remains the preferred treatment for pediatric CD. However, as the majority of pituitary adenomas in pediatric CD are microadenomas or radiologically occult, TSS poses significant technical challenges for neurosurgeons (5).

Here, we present a review of the diagnostic features and surgical outcomes of 22 pediatric CD patients treated at a single center in China over a 20-year period.

## Patients and methods

Between 2002 and 2022, 519 patients underwent TSS for CD performed by a single neurosurgical team in the Department of Neurosurgery, Ruijin Hospital. Twenty-six patients aged 18 years or younger were initially identified as pediatric; four were excluded due to incomplete data or insufficient follow-up. Clinical features of the remaining 22 pediatric patients (10 male, 12 female) were retrospectively reviewed. Mean age at surgery was 15.8 ± 2.5 years (range 9-18), and mean symptom duration prior to diagnosis was 32.0 ± 30.8 months (range 3-108). Mean BMI was 26.4 ± 6.4 (range 18.0-39.7) (**Table 1**). Presenting symptoms included weight gain (18/22), acne (13/22), hirsutism (12/22), moon facies (18/22), striae (19/22), central obesity (10/22), pubertal delay or arrest (4/22), irregular menses (3/12 females), headaches (3/22), visual deficits (2/22), hypertension (7/22), and type 2 diabetes mellitus (2/22) (**Table 2**).

**Table 1:** The demographic information of 22 patients at diagnosis of CD.

No.	Gender	Age	Length of history (ms)	BMI (kg/m^2^)
1	F	18	24	32.72
2	F	18	12	24.24
3	M	14	6	24.28
4	M	18	12	22.32
5	F	18	24	39.7
6	F	15	84	23.47
7	F	16	48	29.88
8	F	18	3	18
9	M	17	108	36.7
10	M	14	17	19.9
11	M	18	36	21.9
12	F	17	6	23.5
13	M	18	72	20.3
14	M	15	24	20.6
15	M	17	96	24.19
16	M	12	24	24.97
17	M	14	24	34.63
18	M	15	3	21.36
19	M	9	18	23.21
20	F	18	24	35.65
21	F	17	36	35.7
22	F	12	3	23.51

**Table 2:** Clinical signs and symptoms of 22 patients at diagnosis of CD.

No.	Acne	Hirsutism	Moon face	Stria	Weight gaining	Central obesity	Headaches	Irregular menses	Vision deficiency	Pubertal delay or arrest	History of diseases		
											Hypertension	T2DM	Others
1	+	–	+	+	+	+	–	–	–	–	+	–	nephrolith
2	+	–	+	+	+	–	+	+	+	–	–	–	–
3		+	+	+	+	–	–	–	–	–	–	–	hepatitis B
4	+	+	+	+	+	+	–	–	–	–	–	–	nephrolith
5	+	–	+	+	+	+	–	–	–	–	–	–	–
6	+	+	+	+	+	+	–	–	–	–	+	–	intrcranial hemorrhage, mucopolysaccharidosis type IV
7	–	–	+	+	+	–	–	–	–	–	–	–	–
8	+	–	+	+		–	–	–	–	–	–	–	–
9	+	+	–	+	+	–	–	–	–	–	–	+	–
10	–	–	+	+	+	+	–	–	–	–	–	–	–
11	–	–	–	+	+	–	+	–	+	–	+	–	–
12	+	+	+	+	–	–	–	+	–	–	–	–	–
13	–	–	+	–	–	–	–	–	–	+	+	–	fundal hemorrhage
14	–	+	–	+	+	+	–	–	–	–	+	–	–
15	+	+	+	+	+	+	–	–	–	+	–	–	–
16	–	+	+	–	+	–	–	–	–	+	–	–	–
17	+	–	+		+	+	–	–	–	–	–	–	–
18	–	+	–	+	–	–	–	–	–	–	–	–	thyroid nodule
19	–	+	+	+	+	–	–	–	–	+	+	–	–
20	+	+	+	+	+	+	+	–	–	–	+	–	–
21	+	–	+	+	+	+	–	–	–	–	–	+	thyroid nodule
22	+	+	+	+	+	–	–	+	–	–	–	–	–

"+" indicates that the patient has the symptom or medical history, while "-" indicates that the patient does not have the symptom or medical history.

Diagnosis of CD was confirmed by a multidisciplinary team comprising radiologists, endocrinologists, interventional radiologists, pediatricians, and neurosurgeons. Clinical manifestations, plasma cortisol circadian rhythm, low-dose dexamethasone suppression test (LDDST, 2 mg dexamethasone), and high-dose dexamethasone suppression test (HDDST, 8 mg dexamethasone) were assessed by pediatricians or endocrinologists. Following the 2mg LDDST, the 48-hour serum cortisol level exceeded 1.8 μg/dL, indicating inadequate suppression. In contrast, after the 8mg HDDST, the 48-hour cortisol level was suppressed to <50% of baseline, demonstrating significant suppression. Bilateral inferior petrosal sinus sampling (BIPSS) with or without desmopressin (DDAVP) stimulation was performed by experienced interventional radiologists. Samples were immediately placed on ice after collection. All biochemical analyses were conducted in a College of American Pathologists-accredited laboratory (No. 7217913).

Preoperative pituitary magnetic resonance imaging (MRI) was performed at 1.5 T or 3.0 T in all patients. T1-weighted and T2-weighted spin-echo images were obtained in coronal and sagittal planes (2-mm slice thickness) before and after gadolinium injection. A dynamic coronal sequence was also acquired within 2 minutes post-injection (**Table 3**).

**Table 3:** Preoperative endocrinological evaluation and neuroimaging results of 22 patients at diagnosis of CD.

No.	Cortisol level (8AM)	Cortisol level (4PM)	Cortisol level (0AM)	24h urine free cortisol	Plasma ACTH (8AM)	HDDST^c^	BIPSS+DDAVP^a^	MRI^b^
1	26.79	21.09	16.25	608.3	64.06	+	L	Mi-L
2	18.43	6.4	15.59	564.97	62.35	+	–	Ma-L
3	35.96	33.97	19.89	1620.27	77.99	–	R	Mi-R
4	20.65	23.07	20.67	724.75	170.05	–	C	Mi
5	16.92	15.47	12.3	347.04	76.1	+	R	Mi
6	29.11	25.29	26.09	619	116.85	+	C	N
7	18.2	15.5	16.9	273.1	40.5	+	–	Mi-L
8	44.63	32.22	29.67	1463	177.5	+	R	Mi-R
9	14.7	14.6	13.5	252.2	124	+	R	Mi-R
10	15.2	24.3	15.1	119.2	154.9	–	R	N
11	24.3	28.3	19.2	457.2	59.2	+	–	Ma-L
12	21.6	16.9	11.9	620.9	83.5	+	R	Mi-R
13	13.8	11.2	14.2	278.5	96.3	+	R	N
14	27.3	21.4	27.8	548.5	95	+	L	Mi-L
15	25.55	21.79	14.18	530.04	54	+	R	Mi
16	18.73	12.26	10.37	230.6	62.13	+	C	N
17	19.28	14.77	22.41	525.14	50.8	+	C	Mi-L
18	26.06	11.78	12.08	995.25	77.9	+	R	Mi-R
19	15.37	11.91	24.71	388.51	12.51	+	R	Mi
20	38.2	23.52	25.99	1726.67	101.3	+	L	Mi-L
21	125.62	79.44	72.25	7669.48	272.6	+	C	Mi
22	21.89	27.32	12.65	979.34	65	+	L	Mi-L

^a^R, laterization of BIPSS at the right side; L, left side; C: No laterization of BIPSS; "-" indicated that BIPSS was not performed. ^b^Mi: microadenoma; Ma: macroadenoma; N: no tumor was demonstrated on MRI. Normal range: 24h urine free cortisol: 21-111μg/24h urine; cortisol level: 6.7-22.6 μg/dl; ACTH: 7–65 pg/ml. ^c^"+" indicates that the result of HDDST is positive, while the "-" means negative.

The same surgical team performed TSS on all patients using a mononostril approach. Microscopic TSS (MTSS) was utilized in 11 patients treated before 2016, while endoscopic TSS (ETSS) was employed in the subsequent 11 patients. For patients with concordant MRI-identified adenomas and BIPSS lateralization, exploration focused on the imaging-identified region, and a rim of pituitary tissue surrounding the tumor cavity was resected. If the tumor involved the cavernous sinus (CS), the inner CS wall was also inspected/explored. If BIPSS lateralization conflicted with MRI findings, the pituitary side indicated by BIPSS was explored first. For MRI-negative tumors, exploration commenced on the side with higher ACTH levels on BIPSS (when available) and proceeded to complete gland inspection. If no adenoma was identified intraoperatively, approximately half of the gland was resected, guided by BIPSS results.

Immediate remission was defined as a postoperative serum cortisol nadir <5 μg/dL or normal 24-hour UFC. Recurrent hypercortisolism was defined as the reappearance of biochemical hypercortisolism after a period of hypocortisolism or clinical adrenal insufficiency. The concordance of BIPSS lateralization with MRI localization refers to whether the tumor side indicated by BIPSS corresponds to the tumor side identified on MRI.

Patients were followed in the outpatient clinic at regular intervals. If endocrine evaluations were performed at local hospitals, results were communicated to the authors via WeChat. Mean follow-up duration was 29.4 months (range 2–129 months).

## Results

Preoperative plasma cortisol levels measured at three time points were: mean 28.10 μg/dL at 8:00 AM (range 14.70-125.62 μg/dL), 22.39 μg/dL at 4:00 PM (range 6.4-79.44 μg/dL), and 20.62 μg/dL at midnight (range 11.9-72.25 μg/dL). Mean preoperative plasma ACTH level at 8:00 AM was 95.21 pg/mL (range 12.51-272.6 pg/mL), and mean 24-hour UFC was 979.18 μg/24h (range 119.20-7669.48 μg/24h). HDDST was positive in 19/22 patients. BIPSS with DDAVP was performed in 19 patients, demonstrating lateralization in 14 patients (4/14 left, 10/14 right).

MRI localized an adenoma in 18/22 patients (81.8%), comprising 16 microadenomas and 2 macroadenomas. Tumor location on MRI was: right sellar (n=5), left sellar (n=8), and central sellar (n=5). Concordance between BIPSS lateralization and MRI localization was 57.89% (11/19).

Immediate postoperative remission was achieved in 20 patients (90.9%). The two patients without immediate remission (Case 2: macroadenoma; Case 6: MRI- and pathology-negative) received additional treatments (Case2: gamma knife radiosurgery; Case 6: ketoconazole). Among the 20 patients with initial remission, 2 (10.0%) experienced recurrence (Case 3: microadenoma; Case 10: MRI-negative). Case3 received pasireotide after recurrence; Case 10 underwent repeat TSS, which did not achieve remission. Subsequent gamma knife treatment also ultimately failed. Ketoconazole therapy was then initiated. The sustained long-term remission rate for the cohort was 81.8% (18/22).

In these cases, intraoperative bleeding was controlled in all cases, and no patient required transfusion. Case 10 experienced a CSF leak following repeat transsphenoidal surgery (TSS). All patients who achieved postoperative remission were administered cortisone replacement therapy. The requirement for levothyroxine replacement differed between groups: one child in the ETSS group (1/11) versus five patients in the MTSS group (5/11). For diabetes insipidus, oral desmopressin was administered to three patients in the ETSS group and two in the MTSS group (**Table 4**).

**Table 4:** The neurosurgical outcome and follow-up results of 22 patients of CD.

No	Operation	Nadir cortisol level after operation	Pathology^a^	Remission	Hormonal replacement	Diabetes insipidus^b^	Recurrent	Additional therapy
1	ETSS	2.39	+	R	Cortisol	+		
2	ETSS	17.28	+	U				γ-knife
3	ETSS	1.69	+	R	Cortisol		+^1^	pasireotide
4	TSS	2.07	+	R	Cortisol+ Euthyrox			
5	TSS	0.34	+	R	Cortisol	+		
6	TSS	25.45	-	U	Euthyrox			ketoconazole
7	TSS	0.73	+	R	Cortisol+ Euthyrox			
8	TSS	2.7	+	R	cortisol			
9	TSS	1.4	+	R	Cortisol+ Euthyrox			
10	TSS	4.05	+	R	cortisol		+^2^	re-TSS+γ-knife+ketoconazle
11	TSS	3.4	+	R	cortisol			
12	TSS	1.6	+	R	cortisol			
13	TSS	2.1	+	R	cortisol			
14	TSS	1.9	+	R	Cortisol+ Euthyrox	+		
15	ETSS	1.6	+	R	cortisol			
16	ETSS	1.4	+	R	cortisol	+		
17	ETSS	1.21	+	R	cortisol			
18	ETSS	2.23	+	R	cortisol			
19	ETSS	1.49	+	R	Cortisol+ Euthyrox	+		
20	ETSS	1.3	+	R	cortisol			
21	ETSS	2.89	+	R	cortisol			
22	ETSS	1.13	+	R	cortisol			

Recurrence time: ^1^38 months after ETSS; ^2^29 months after TSS. ^a^"+" indicated that pituitary adenoma was confirmed by pathology; "-" indicated that no pituitary adenoma was confirmed. ^b^"+" indicated that diabetes insipidus was happened after surgery.

## Discussion

This 20-year single-center experience represents one of the largest reported cohorts of surgically managed pediatric Cushing’s disease patients. Our findings demonstrate that transsphenoidal surgery (TSS), whether microscopic (MTSS) or endoscopic (ETSS), is a highly effective first-line treatment for pediatric CD, achieving an immediate remission rate of 90.9% and a sustained long-term remission rate of 81.8%.

The diagnostic complexity of pediatric CD is highlighted by the significant diagnostic delay observed (mean 32.0 months) and the occurrence of MRI-negative cases (4/22, 18.2%). This aligns with established literature emphasizing the challenges of pediatric CD diagnosis stemming from its rarity, atypical presentation, and the high proportion of microadenomas or radiologically occult tumors (3, 4, 6–8). Our adherence to a rigorous multidisciplinary diagnostic protocol, incorporating standardized biochemical testing (LDDST, HDDST), BIPSS with DDAVP stimulation (performed in 19/22), and high-resolution dynamic pituitary MRI, reflects current best practices for confirming ACTH-dependent Cushing’s syndrome and tumor localization. The moderate concordance rate (57.89%) between BIPSS lateralization and MRI localization underscores their complementary roles, particularly in cases with equivocal imaging. BIPSS remains critical for guiding surgical exploration in MRI-negative or discordant cases, as evidenced by its use in our decision-making algorithm (9, 10).

Our immediate remission rate (90.9%) compares favorably with contemporary pediatric CD surgical series, which typically report rates between 70% and 98% (1–3, 8, 11–13). The two immediate surgical failures occurred in patients with a macroadenoma (Case 2) or an MRI- and pathology-negative diagnosis (Case 6), profiles consistently associated with lower remission rates. The long-term remission rate of 81.8% (18/22) is robust, although the recurrence rate of 10% (2/20 initially remitted patients) merits attention. Both recurrences arose in patients with microadenomas, one of whom was MRI-negative (Case 10). This recurrence rate falls within the reported range (5-30%) for pediatric CD and reinforces the need for lifelong endocrine surveillance (1, 14, 15). The relatively short mean follow-up (29.4 months) suggests that the true recurrence rate might be higher with extended observation, representing a limitation of this study.

Our experience reflects the evolution of surgical technique, with a transition from MTSS to ETSS after 2016. While the cohort size and follow-up duration preclude definitive conclusions regarding the comparative efficacy of MTSS versus ETSS in this specific pediatric population, both techniques yielded high success rates. In our group, no significant differences exist in remission or recurrence rates. However, regarding complications, ETSS demonstrates a lower incidence of hypopituitarism compared to MTSS, while the incidence of diabetes insipidus is similar. It should be noted, however, that this comparison remains limited by the small number of reported cases. The endoscopic approach offers theoretical advantages, such as wider panoramic visualization potentially aiding in the identification of small or laterally extending microadenomas, which are common in children. Larger, prospective studies with longer follow-up are warranted to directly compare outcomes between these surgical modalities in pediatric CD.

The spectrum of clinical manifestations observed (e.g., weight gain, moon facies, striae, hypertension, pubertal arrest/delay) demonstrates the profound multisystem impact of hypercortisolism in children. The notable prevalence of metabolic complications like hypertension (7/22) and type 2 diabetes mellitus (2/22), even in this young cohort, highlights the urgency of timely diagnosis and effective intervention to mitigate long-term morbidity (5, 16–18).

## Limitations

This study shares the limitations inherent to retrospective, single-center designs. The modest sample size, though substantial for this rare condition, limits statistical power for subgroup analyses, such as rigorous comparison of MTSS vs. ETSS outcomes or identification of specific predictors of failure/recurrence. The mean follow-up period is relatively short for a disease with potential for late recurrence, long-term remission rates may be lower than reported, and the study could not capture long-term complications of TSS, particularly those affecting growth and development in pediatric patients. Detailed data on specific postoperative complications (e.g., diabetes insipidus, hypopituitarism) and pituitary function during follow-up would provide a more comprehensive assessment of treatment sequelae but were not the primary focus of this outcome report.

## Conclusion

Despite the inherent diagnostic and therapeutic challenges of pediatric Cushing’s disease, transsphenoidal surgery performed in a specialized center achieves high rates of immediate and sustained remission. Our results support the efficacy of TSS as the primary treatment modality. A meticulous multidisciplinary diagnostic approach, including BIPSS when indicated, is crucial for success, particularly in MRI-negative cases. While recurrence remains a concern necessitating vigilant long-term follow-up, the majority of children with CD can attain durable disease control with appropriate surgical management. The transition to endoscopic techniques proved safe and effective, warranting further investigation in larger pediatric cohorts with extended follow-up.

## Data Availability

The original contributions presented in the study are included in the article/supplementary material. Further inquiries can be directed to the corresponding authors.
